# Supplementary material for: Evaluation of a very brief pedometer-based physical activity intervention delivered in NHS Health Checks in England: The VBI randomised controlled trial
Source: PLoS Med. 2020 Mar 6;17(3):e1003046. doi: 10.1371/journal.pmed.1003046 (PMC7059905; doi:10.1371/journal.pmed.1003046)
Supplement: S1 Text — (PDF) [file pmed.1003046.s001.pdf]

Statistical Analysis Plan
for the VBI trial

The VBI trial: A randomised controlled trial of the efficacy and cost-effectiveness of a very brief intervention to increase physical activity in adults attending NHS Health Checks

SAP VERSION	2.0
ISRCTN NUMBER	72691150
SAP VERSION DATE	14/03/2016
TRIAL ANALYSIS STATISTICIAN	Ms Joana Vasconcelos
TRIAL CHIEF INVESTIGATOR	Professor Stephen Sutton
SAP AUTHORS	Ms Joanna Mitchell<br>Professor Toby Prevost<br>Dr Ed Wilson

1 Table of Contents

1 Table of Contents 2

2 Abbreviations and Definitions 4

3 Introduction 4

3.1 Preface 4

3.2 Purpose and Scope of the Statistical Analysis Plan 6

4 Study Objectives and Endpoints 8

4.1 Study Objectives 8

4.2 Endpoints 8

4.3 List of Outcome Measures 8

4.4 Derived Variables 9

5 Study Methods 10

5.1 General Study Design and Plan 10

5.2 Randomisation and Blinding 11

5.3 Inclusion/Exclusion Criteria 12

5.4 Event Schedule 13

6 Study Variables 13

6.1 Baseline descriptive variables (collected in the self-administered questionnaire at baseline). 14

6.2 Questions on Health Check 17

6.3 NHS use, time off work and expenditure on physical activity Questionnaire 18

6.5 RPAQ 22

7 Sample Size 22

8 General Considerations 23

8.1 Timing of Analyses 24

8.2 Analysis Populations 24

8.3 Covariates and Subgroups 25

8.4 Missing Data 25

8.4.1 Adjustment of Confidence Intervals and p-value 25
9 Summary of Study Data 26
9.1 VBI Consort Statement 27
9.2 Baseline comparability of randomised groups 28
9.3 Comparison of rates of withdrawal and losses to follow up 28
9.4 Comparison of those followed-up with those lost to follow up 28
10 Statistical Analyses 28
10.1 Primary Efficacy Analyses 29
10.2 Secondary Efficacy Analyses 29
10.3 Economic Analyses 30
11 Figures 31
12 Reporting Conventions 31
13 Technical Details 31
14 References 32

2 Abbreviations and Definitions

AE	Adverse Event
CRF	Case Report Form
PA	Physical Activity
SAP	Statistical Analysis Plan
VBI	Very Brief Intervention
ITT	Intention to Treat
PP	Per Protocol

3 Introduction

3.1 Preface

Vascular disease, which includes coronary heart disease, stroke, diabetes and kidney disease, affects more than four million people in England, causes one out of three deaths and one out of five hospital admissions [1]. Unhealthy behaviours such as a sedentary lifestyle are important risk factors for these diseases [1], and lack of physical activity is the fourth most important risk factor worldwide [1]. In addition, physical inactivity in the UK represents a direct and indirect cost to society of £8.2 billion [2]. The current recommendation for adults is that they should aim to take 30 minutes of moderate intensity physical activity, e.g., brisk walking, on at least five days per week. The 2008 Health Survey for England [3] found that only 44% of men and 33% of women between 35 and 44 years met this recommendation. This fell to 20% of men and 18% of women aged 65 to 74 years. These figures are based on self-reports of physical activity, and people tend to overestimate how active they are. When activity was measured objectively with an accelerometer, only 6% of men and 4% of women met the recommendation. Despite the hoped for Olympics Legacy Effect, initial results from the 2012 Health Survey for England [3] indicate that these figures have remained stable.

There are several systematic reviews of the effects of brief interventions to promote physical activity. A narrative review by NICE in 2006 concluded that brief advice by primary care practitioners showed promise, but there was uncertainty about the most effective approaches among groups at higher risk of chronic disease [4]. The US Preventative Services Task Force stated that it was not clear whether physical activity

counselling in primary care is effective and they recommended more research on this topic [5]. Published trials of physical activity interventions suffer from a number of limitations. The interventions are often not described in sufficient detail to identify the 'active ingredients' and to enable others to replicate the intervention [6, 7], and many trials use only self-report measures of physical activity, which may lack validity. The recent NICE report [8] focuses on brief physical activity advice and acknowledges that brief advice has some impact on physical activity levels; however, there is insufficient evidence to make recommendations about the differential impact of brief advice based on duration of delivery, content or by who delivers it. However, health professionals have been identified as having the potential to be powerful mediators for physical activity promotion [9], and the US Preventive Service Task Force more recently state [10] that behavioural interventions may be more effective when undertaken in the context of broader public health interventions. However, for some health professionals, time (e.g. limited consultation time) and a lack of perceived efficacy (i.e. doubt about whether the intervention will lead to an increase in physical activity) are seen as key barriers to the success of such interventions [9].

The NHS is currently conducting Health Checks which are offered to all adults between 40 and 74 years. Health Checks include an assessment of a person's risk of vascular disease and advice and treatment tailored to the person's individual need [1]. Public Health England [PHE] fully supports the NHS Health Check programme [11] and works with local authorities to improve the uptake of this scheme. The Health Checks therefore offer an ideal opportunity to deliver very brief physical activity interventions to a large proportion of the population. Very brief physical activity interventions (lasting no more than 5 minutes) should be relatively easy and inexpensive to implement on a large scale, and a small effect on physical activity level could translate into a significant public health benefit.

We have developed several very brief interventions and have conducted pilot studies, including a pilot trial (12-EE-0200), to assess their feasibility, acceptability and potential efficacy when delivered by a practice nurse or healthcare assistant as part of an NHS Health Check. This randomised controlled trial (RCT) will test the cost and efficacy of the intervention judged to be the most promising (i.e. it can be delivered within 5 minutes and is acceptable, feasible and potentially effective) in promoting an increase in physical activity, namely a pedometer-based intervention. The comparator will be a routine Health Check (which includes basic lifestyle advice). The

trial will thus estimate the incremental efficacy and cost effectiveness of adding a very brief pedometer-based physical activity intervention to the routine Health Check.

3.2 Purpose and Scope of the Statistical Analysis Plan

The purpose of this statistical analysis plan is to set out the study objectives and hypotheses of the VBI Trial and the analytical approaches and procedures necessary to address these. It is expected that this document will inform the statistical methods section of the trial paper and provide guidance for further research based on consistent approaches to analysis and methods selected for analysis.

The plan draws on statistical guidance from the ICH Harmonised Tripartite Guideline: Statistical Principles for Clinical Trials (1998), the CONSORT statement for reporting trials (2010), and the Good Clinical Practice Guide (2012) compiled by the MHRA.

Analysis will be conducted once all patients have been followed up for 3 months. Participants will be classified as 'lost to follow up' once every effort has been exhausted to retrieve follow up data.

Participant recruitment is expected to continue until autumn 2015.

Figure 1 shows the expected time frame for the VBI trial.

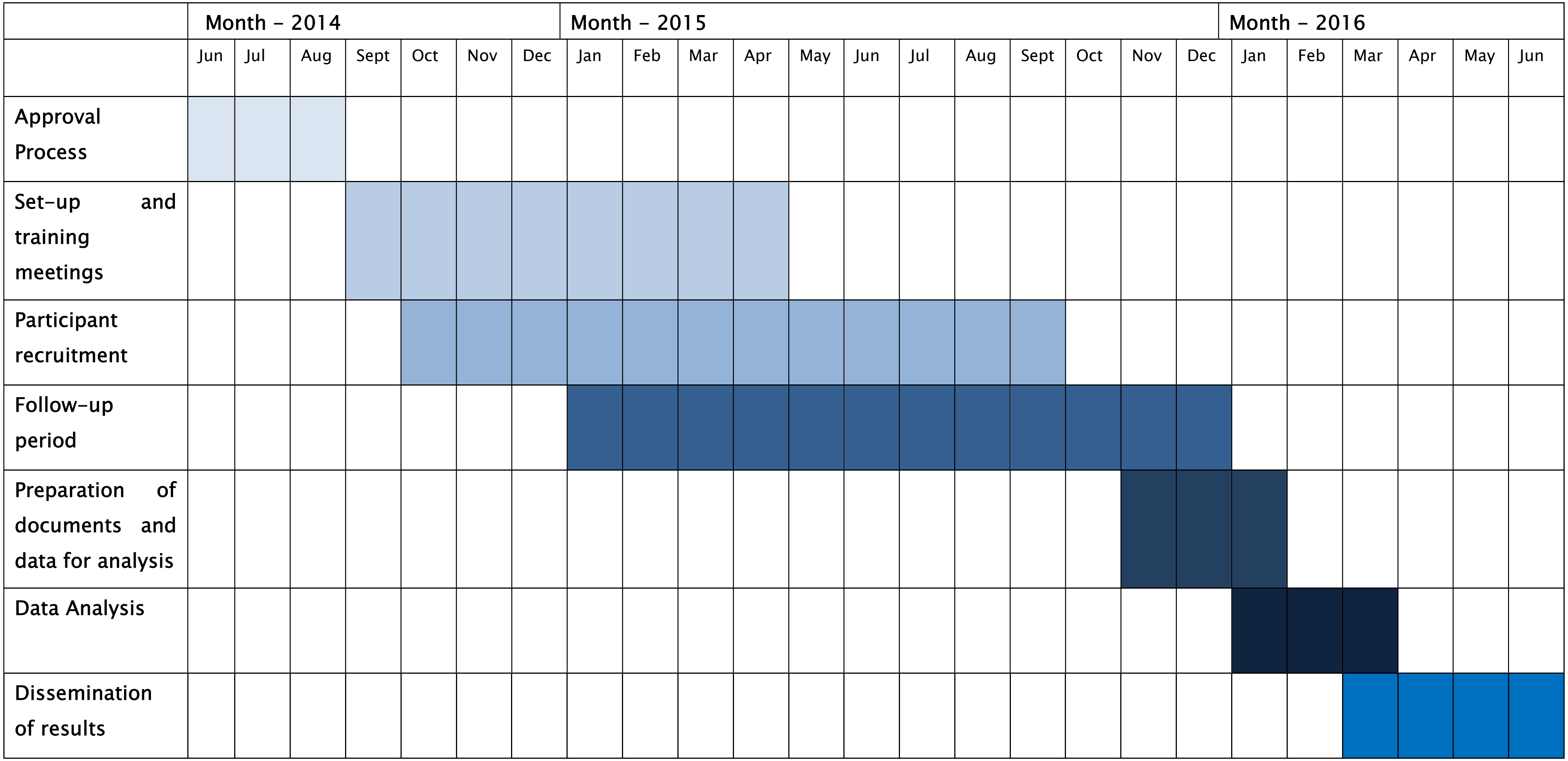

Figure 1: Time frame for the VBI Trial

4 Study Objectives and Endpoints

4.1 Study Objectives

The objective of this trial is to evaluate the effectiveness and cost-effectiveness of a very brief pedometer-based intervention to promote physical activity in adults aged 40-74 years attending an NHS Health Check.

4.2 Endpoints

The primary outcome is physical activity counts per minute at 3-month follow-up, as measured objectively by total acceleration per day.

The secondary outcomes are: Other objectively measured activity related outcomes, Self-reported physical activity, as measured by the Recent Physical Activity Questionnaire and cost, as measured by the Resource Use Questionnaire.

4.3 List of Outcome Measures

Outcome Measure	Baseline (B) /Follow up (FU)	Continuous/categorical
Activity Counts per minute (primary outcome measure)	FU	Continuous
Step Counts	FU	Continuous
Time (min/day) in moderate or vigorous activity	FU	Continuous
Time (min/day) in vigorous [illegible]	FU	Continuous
Time (min/day) in moderate activity	FU	Continuous
PAEE Physical activity energy expenditure (kj/kg/day)	FU	Continuous
Home based PAEE (kj/kg/day)	FU	Continuous
Work based PAEE (kj/kg/day)	FU	Continuous

Leisure based PAEE (kj/kg/day)	FU	Continuous
Commuting PAEE (kj/kg/day)	FU	Continuous
Screen/TV time (hours per day)	FU	Continuous
10 year cardio-vascular risk score (Q Risk)	B	Continuous

4.4 Derived Variables

<u>Physical Activity variables derived from accelerometer data:</u>

Time-series accelerometer data will be processed, accounting for non-wear time. Data will be summarised into hourly, daily and summary level information reflecting overall activity levels as well as time spent at different acceleration intensity levels.

As for the pilot trial, vector magnitude (VM) versions of accelerometer-derived measures will be used, including for the primary outcome counts per minute, and for the time spent in acceleration intensity levels. The latter will be defined as "Light" (0 to 2689 counts, using VM), "Moderate" (2690 to 6166 counts, using VM), and "Vigorous but not Very Vigorous" (>6166 - 9642 counts, using VM), and "Very Vigorous" (>9642), each measuring the length of time in minutes in that intensity category. "Vigorous" is then derived as the sum of the minutes of time spent in "Vigorous but not Very Vigorous" and that spent in "Very Vigorous" (i.e. >6166 counts, using VM). It is not intended to report the "Very Vigorous" outcome in isolation in the main trial because the prevalence and duration in this category were much too low to allow any meaningful analysis in the pilot trial. The total time in moderate or Vigorous activity will be reported, and this is defined using a threshold of 2690 counts, using VM.

Resource items associated with the intervention comprise training for nurses in how to deliver the intervention, the time required to deliver the intervention to patients, printed materials and a pedometer. Timings and costings will be extracted from trial records.

NHS primary and secondary resource use quantities will be extracted from the patient-completed resource use questionnaire, questions 1 & 2. Costs will be calculated by multiplying quantities of resources by unit costs extracted from

routine NHS sources including Curtis et al. and the NHS Reference Costs database. All unit costs will be from a common price year, or where unavailable, prices from alternative years converted to the price year of the analysis using the HCSC inflation index. The price year will be determined at the point of analysis and will be the latest for which unit cost data are available.

Out of pocket costs will comprise expenditure associated with health or sports clubs, including travel as reported by respondents (resource use questionnaire, questions 3-10). Reported costs will be adjusted to the price year of the analysis using the CPI inflation index.

The cost of absenteeism and presenteeism will be calculated using a modified version of the Work Productivity and Activity Impairment Questionnaire (Reilly, Zbrozek & Dukes 1993) (Questions 11-16 of the resource use questionnaire).

Physical activity variables derived from items in the RPAQ questionnaire:

For latest information on cleaning and processing go to:

http://www.mrc-epid.cam.ac.uk/research/resources/materials-transfer-disclaimer/physical-activity-downloads/

Individual Deprivation Score:

Calculated by scoring one point for each of the following: no qualifications; unemployed or full-time student; renting their home; no cars. We will derive an area-based index of deprivation (IMD; Index of Multiple Deprivation) from the postcode recorded o the consent form.

5 Study Methods

5.1 General Study Design and Plan

The design is a two parallel group randomised controlled trial (RCT) with individual allocation comparing usual care (standard Health Check) with usual care plus a very brief pedometer-based intervention.

The control condition in the VBI trial is the standard Health Check. Variations in how this is delivered across GP practices will be described.

The Pedometer Intervention will be delivered at the end of the Health Check and consists of the following three components.

Face-to-face discussion in which the practitioner:

- gives the patient feedback on their current activity;
- gives information about the current physical activity recommendations (30minutes of moderate-intensity activity on 5 or more days a week OR 10,000 steps per day);
- shows the patient how to wear/use the pedometer and encourages them to use it to monitor the number of steps walked each day;
- explains that the Pedometer Booklet gives tips for how to increase daily steps by making small changes;
- shows the patient the Step Chart and encourages them to use it to set a step goal and record daily steps to monitor whether they reached that goal.

Pedometer & Step Chart: A Yamax Digiwalker SW200 and a printed Step Chart are given to the patient to use as self-monitoring tools.

Pedometer Booklet: containing information on the UK government physical activity recommendations, instructions on how to use the pedometer, and tips for achieving more steps.

Neither researcher nor participant will be blinded to treatment group.

5.2 Randomisation and Blinding

Randomisation to trial group will be carried out by the nurse or healthcare assistant at the beginning of the Health Check using a web-based randomisation tool called Sealed Envelope (http://www.sealedenvelope.com). The stratification by practitioner will use blocks of sufficiently small size to provide effective balance across arms.

5.3 Inclusion/Exclusion Criteria

Inclusion Criteria

Participants will be adults aged 40-74 who are eligible to be invited for a NHS Health Check and who are able to give consent for participation in the trial. Patients are eligible for a NHS Health Check if they have not already been diagnosed with vascular disease such as type 2 diabetes, heart disease, kidney disease and peripheral vascular disease and who are not already on a separate care pathway for identifiable risks such as raised blood pressure. Non-English speaking patients may bring with them an interpreter to aid with the understanding of the Health Check and the trial or (if it is available) use the translator facility provided by the GP Surgery. It will be made clear however that for a patient to be included in the trial they must be able to complete the follow up measurements 3 months after the Health Check for which an interpreter may still be required. Any patient unable to fulfil this criterion is still eligible for the NHS Health Check.

Exclusion Criteria

The GP practice will exclude patients they consider unsuitable for the trial (i.e. any patient where receiving an invitation will cause unnecessary stress due to mental health issues or a diagnosis of terminal illness for example).

5.4 Event Schedule

GP: Identification of eligible participants

Research team: Random selection of eligible patients
The list of randomly selected patients will be fed back to the GP practice.

GP Surgery: Recruitment
GP surgeries? invite patients for a Health Check and to take part in the VBI trial.
Participants will make an appointment with their surgery for a Health Check mentioning at the time of booking that they are interested in taking part in the VBI trial.

GP Surgery: The Health Check
Consent is obtained at the start of the consultation by the nurse or healthcare assistant doing the Health Check.
(Nurse or HCA will randomise the patient to either the Intervention or the Control group using a web-based tool)
Deliver the Health Check with or without the intervention (audio-record if required)
Completion of Case Report Form

Research team: Follow-up at 3 months
Research team send a reminder text/email 1 week befor[illegible] sending the accelerometer and questionnaires.
Researcher sends out an accelerometer with instructio[illegible] and 2 questionnaires. All participants who complete w[illegible] be entered into a prize draw.

Research team/GP surgery: Medical Records
Surgery database searched for 10 year cardio-vascular risk score calculated at the Health Check for each participant.

VBI Invitation pack:

Participant Information Sheet

Invitation Letter

Sample Consent

Participants complete a short questionnaire

Participants wear accelerometer for a week and return to research team in a freepost envelope with completed questionnaires

6 Study Variables

Baseline physical activity status is recorded on the Case Report Form. Physical activity is rated by the nurse or healthcare assistant doing the health check and is derived from the template used during the Health Check.

The duration of follow up will be a minimum of 3 months after the Health Check; up until either the follow up measures are collected or the participant is recorded as lost to follow up. An accelerometer and 2 questionnaires will be sent to participants about 3 months after their Health Check although the data on some participants may have to be collected later (due to participant holidays, illness or other reasons). Information on patient characteristics is collected at baseline, self-reported physical activity and NHS use, time off work and expenditure on physical activity is collected at follow-up. Objective physical activity data will be obtained from the accelerometers worn for approximately one week.

6.1 Baseline descriptive variables (collected in the self-administered questionnaire at baseline).

Item number	Description	Variable name	Range/code
1	Date of birth	DoB	DD/MM/YYYY
2	Date of questionnaire completion	DoCom	DD/MM/YYYY
3	Gender	Sex	1. Male<br>2. Female

4	Ethnicity	Ethnic	15 categories<br><br>1. White British<br>2. White Irish<br>3. Other white<br>4. Mixed black<br>5. Mixed Asian<br>6. Other mixed<br>7. Asian – Indian<br>8. Asian – Pakistani<br>9. Asian – Bangladeshi<br>10. Other Asian
5	Qualifications	Qualif	5 categories<br><br>1. None<br>2. GCSE<br>3. A-level<br>4. Degree<br>5. Other
6	Work Status		1. Paid work<br>2. Homemaker<br>3. Unemployed<br>4. Full-time student<br>5. Retired
7	Occupation Activity		1. Manual<br>2. Non-manual<br>3. Other
8	Income		1. less than £18,000<br>2. £18,000 – £30,999<br>3. £31,000 – £51,999<br>4. £52,000 – £100,000<br>5. Greater than £100,000

9	Marital Status	MarStatus	5 categories:<br>1. Single<br>2. Married<br>3. Divorced<br>4. Separated<br>5. Widowed<br>6. Co-habiting
10	Home Ownership		1. Own outright<br>2. Own with mortgage<br>3. Rent from private landlord<br>4. Rent from local authority<br>5. Part rent/part mortgage<br>6. Other
11	Vehicles Owned		1. Yes<br>2. No
12	Dependents		1. Yes<br>2. No

6.2 Questions on Health Check (collected at follow-up)

Item number	Description	Variable name	Range/code
1	PA mentioned in HC?	PAinHC	1. No<br>2. Yes
2	Given advice?	GivenAdvice	1. No<br>2. Yes
3	Worn the pedometer?	WornPed	1. No<br>2. Yes
4	Recorded step count?	RecStepCount	1. No<br>2. Yes
5	Set step goals	SetGoals	1. No<br>2. Yes
6	Thought about benefits of PA?	PABenefits	1. No<br>2. Yes
7	Do you know the PA recommendations?	PARecommendations	1. No<br>2. Yes
8	Become more aware of PA?	PAMoreAware	1. No<br>2. Yes
9.	Given a booklet?	Booklet	1. No<br>2. Yes
10	If Yes,<br>a) Read booklet?<br>b) Still have the booklet?	ReadBooklet<br>KeptBooklet	1. A) No<br>2. A) Yes<br>1. B) No<br>2. B) Yes
11	Do you know other people taking part in the trial?	OtherPeople	1. No<br>2. Yes
12	If Yes were they in the Intervention Group (A) or the Control group (B)	YesIntOrControl	A<br>B

6.3 NHS use, time off work and expenditure on physical activity Questionnaire (collected at follow-up)

Item number	Description	Variable name	Range/code
1	See GP or practice nurse?	SeeGPNurse	1. No<br>2. Yes
2.	Number of GP surgery appointments	GPSurgery	Integer
3	Number of GP home visits	GPHome	Integer
4	Number of GP phone consultations	GPPhone	Integer
5	Number of nurse surgery appointments	NurseSurgery	Integer
6	Number of nurse home visits	NurseHome	Integer
7	Number of nurse phone consultations	NursePhone	Integer
8	Number of other surgery appointments	OtherSurgery	Integer
9	Number of other home visits	OtherHome	Integer
10	Number of other phone consultations	OtherPhone	Integer
11	Other type	OtherType	Free text

12	Been into hospital?	AttendHospital	1. No<br>2. Yes
13	Outpatient visits	OutpatientsNumber	Integer
14	Outpatient reason for visit(s)	OutpatientReason	Free text
15	Day case procedures	DaycaseNumber	Integer
16	Day case reason for visit(s)	DaycaseReason	Free text
17	Inpatient stays	InpatientNumber	Integer
18	Inpatient reason for visit	InpatientReason	Free text
19	A&E attendances	AandENumber	Integer
20	A&E reasons	AandEReasons	Free text
21	Other attendances description	OtherAttendDescript	Free text
22	Other attendances number	OtherAttendNumber	Integer
23	Other attendances reasons	OtherAttendReasons	Free text
24	Inpatient length of stay	InpatientLength	Integer
25	Membership of health clubs etc.	HealthClubMember	1. No<br>2. Yes

26	Travel to health club etc.	HealthClubTravel	1. Walk/cycle<br>2. Hospital/community transport<br>3. Car<br>4. Public transport<br>5. Taxi<br>6. Park&Ride
27	Travel to health club other	HealthClubTravelOther	Free text
28	Cost of fares and/or parking	CostOfTravel	Single
29	Travel mileage	Mileage	Single
30	# visits per week to health club etc.	HealthClubVisits	Integer
31	Time spent to attend health club etc.	HealthClubTime	hhmm
32	Details of fitness centre memberships	HealthClubDetails	Free text (as below)
33	Expenditure over last 3 months	HealthClubCosts	single (These will need a separate table in a relational database as could be repeated several times)
34	Details of other out of pocket expenditure	OtherDetails	Free text
35	Out of pocket expenditure	OtherCosts	Single (as above)

36	In Employment	Employed	1. No<br>2. Yes
37	Hours off work in last 7 days due to ill health	MissWorkHealth	hhmm
38	Hours off work due to other reason (e.g. holiday)	MissWorkOther	hhmm
39	Hours worked in last 7 days	HoursWorked	hhmm
40	How much have health problems affected productivity in last 7 days whilst working	WorkProductivityProbs	Integer 0-10
41	How much have health problems affected regular activities other than work	DailyActivityProbs	Integer 0-10

6.5 RPAQ

For the latest information on the RPAQ go to

http://www.mrc-epid.cam.ac.uk/research/resources/materials-transfer-disclaimer/physical-activity-downloads/

7 Sample Size

A trial of 394 followed up per arm is sufficient to detect a 0.2sd ("small") difference in mean activity between groups (40 accelerometer counts per minute) with 80% power. Allowing for attrition of 30% at follow-up (i.e. 30% of participants not providing sufficient accelerometer data), an initial sample size of 570 per group (and so a trial total sample size of 1140) would give 80% power to detect an effect of this size between the two conditions (alpha = 0.05, 2-sided test).

Observations from our pilot trial (12-EE-0200) suggest that a trial of this size is feasible within the specified resources and timeframe, given the large numbers of people who will be invited for NHS Health Checks, the brevity of the intervention, the remote measurement (done by post), and a 3-month follow-up period. Recruitment to the pilot trial varied depending on the time of year (e.g. flu clinics reduced the time available for Health Checks) but recruitment rate per practice averaged two participants per week. Therefore assuming that each practice recruits two participants a week this would yield about 50 participants per practice over a 6-month period. We would therefore need 23 practices to give the required sample size of 1140.

8 General Considerations

8.1 Data management

The administrative database is managed in-house. The database has been designed to allow easy entry of Case Report Form data and the participant questionnaire. Random checks are performed on the entered data against paper records and all errors are logged and corrected.

Double data entry of the follow up questionnaire is done by an experienced independent agency. The resulting spreadsheets will be interrogated for outliers and corrected. In addition random checks are applied as above.

The objective PA data is checked for completeness and participants will be asked to wear the Actigraph monitor for extra days if the minimum wear time has not been achieved.

All audio recordings are submitted to an independent transcription agency and stored on a secure drive on University computers.

8.2 Timing of Analyses

The final analysis will be performed after all the follow up data has been collected. There will be no interim analysis.

8.3 Analysis Populations

The intention to treat population comprises all patients that have been randomised, regardless of finding any later to have been ineligible or any controls who have mistakenly received the behavioural intervention, or any intervention arm participants who have not received the behavioural intervention.

The per protocol population comprises all participants except those who are later found to have been ineligible or who have not received their randomised intervention (a crosscheck between the randomisation records, CRF and the follow up questionnaire will determine those that have not received their randomised intervention).

The primary efficacy population is the ITT population comprising all randomised patients. The per protocol population is secondary.

An intention to treat strategy [12] will be used in which two analyses will be undertaken. The first analysis will omit participants with missing data on a per outcome basis. This implicitly assumes that the outcome data is missing at random conditional on the covariates in the analysis model. In the absence of baseline physical activity data to be able to informatively impute missing outcome data, a second, sensitivity, analysis will examine the influence of the missing at random assumption on the primary results.

Therefore the primary analyses will be based on a reduced subset of the randomised participants, but the sensitivity analyses will involve all participants. This is why this set of two analysis forms an intention to treat strategy [12].

Inclusion in the primary analyses will be clearly defined according to whether sufficient outcome data is provided by the participant. There will be no other reasons for exclusion. From the pilot trial, the primary outcome is valid and sufficient for analysis if the participant provides a minimum of 3 days of activity data on which a minimum of 10 hours of wear-time data is provided, regardless of how many of the provided days are weekend days or weekdays.

8.4 Covariates and Subgroups

Randomisation is of individuals within each practice rather than cluster randomisation of whole practices to trial arm. The absence of cluster randomisation means that a practice-specific effect will be present in both trial arms, and so general practice will not need to be included as a random effect to reflect increased variation arising from cluster randomisation. In the analysis models, it is intended that general practice will be included as a fixed effect for continuous outcomes as general practice is a stratifier of the randomisation and also the sample size is sufficiently large to support general practice for continuous outcomes. This will be reviewed if the number of stratification categories needs to be increased during the trial. It is not intended that general practice ? be included in the analysis of those outcomes in the trial that are categorical. Although the pilot trial did not indicate significant differences in the primary outcome by gender or age, gender and five-year age-group will be included in the analysis of continuous outcomes so that any confounding effects arising from differences in these variables by arm, and by dropout status, will be adjusted for within the main analyses.

Any subgroup variables documented in the protocol will be analysed using an interaction between subgroup variable and trial arm in order to compare the controlled intervention effectiveness between subgroups for the primary outcome alone. P-values will be used for comparisons between subgroups. Randomised intervention effects within categories of the subgroup variables will be further summarised using 95% confidence intervals.

8.5 Missing Data

As indicated above, a complete case analysis with adjustment for covariates (gender and five-year age-group for all outcomes, and general practice for continuous outcomes) will be adopted along with sensitivity analysis for the primary outcome. This combination represents an Intention to Treat Strategy.

8.5.1 Adjustment of Confidence Intervals and p-value

A 5% significance level will be used for the analysis of the single pre-specified primary outcome. All tests will be two-sided. Secondary outcomes and subgroup analyses will also be assessed at the 5% level of significance. Caution will be taken when

interpreting the analyses of secondary outcomes and subgroup analyses, in order that the possibility of Type 1 errors is recognised. In the absence of any interim analysis, there will be no need to adjust the level.

9 Summary of Study Data

An initial table of baseline characteristics by arm will demonstrate the comparability of the trial arms at baseline, reporting categorical variables (such as gender, and employment status) as percentages (with numerator and denominator) and continuous variables (such as age) as mean with standard deviation or median with interquartile range.

Tables will be used to show between-arm differences in outcomes at follow up. Statistics to be used will be adjusted means with 95% confidence intervals for continuous outcomes. The arithmetic mean will be used for most outcomes, whereas the geometric mean will be used for skewed outcomes where a log transformation will improve the consistency of model residuals with a normal distribution. Based on the pilot trial, this is anticipated for some of the physical activity variables such as time in vigorous activity. Statistics for arm comparisons will be the adjusted difference in arithmetic means with 95% confidence interval, and the percentage increase or? decrease in the intervention group's geometric means compared to that in the control group. For severely skewed outcomes, potentially with influential outliers, a nonparametric Bootstrap approach will be considered in order to provide a reliable confidence interval. Any binary outcomes will be summarised using proportions and adjusted odds ratios.

In the tables, the Control arm will appear to the left of the Intervention arm.

We will use a coding framework to analyse the voice recordings. Features of the Health Check and the Intervention will be coded on a Yes-present/No-absent basis to provide a descriptive analysis of the Health Check and the VBI across and between GP practices.

9.1 VBI Consort Statement

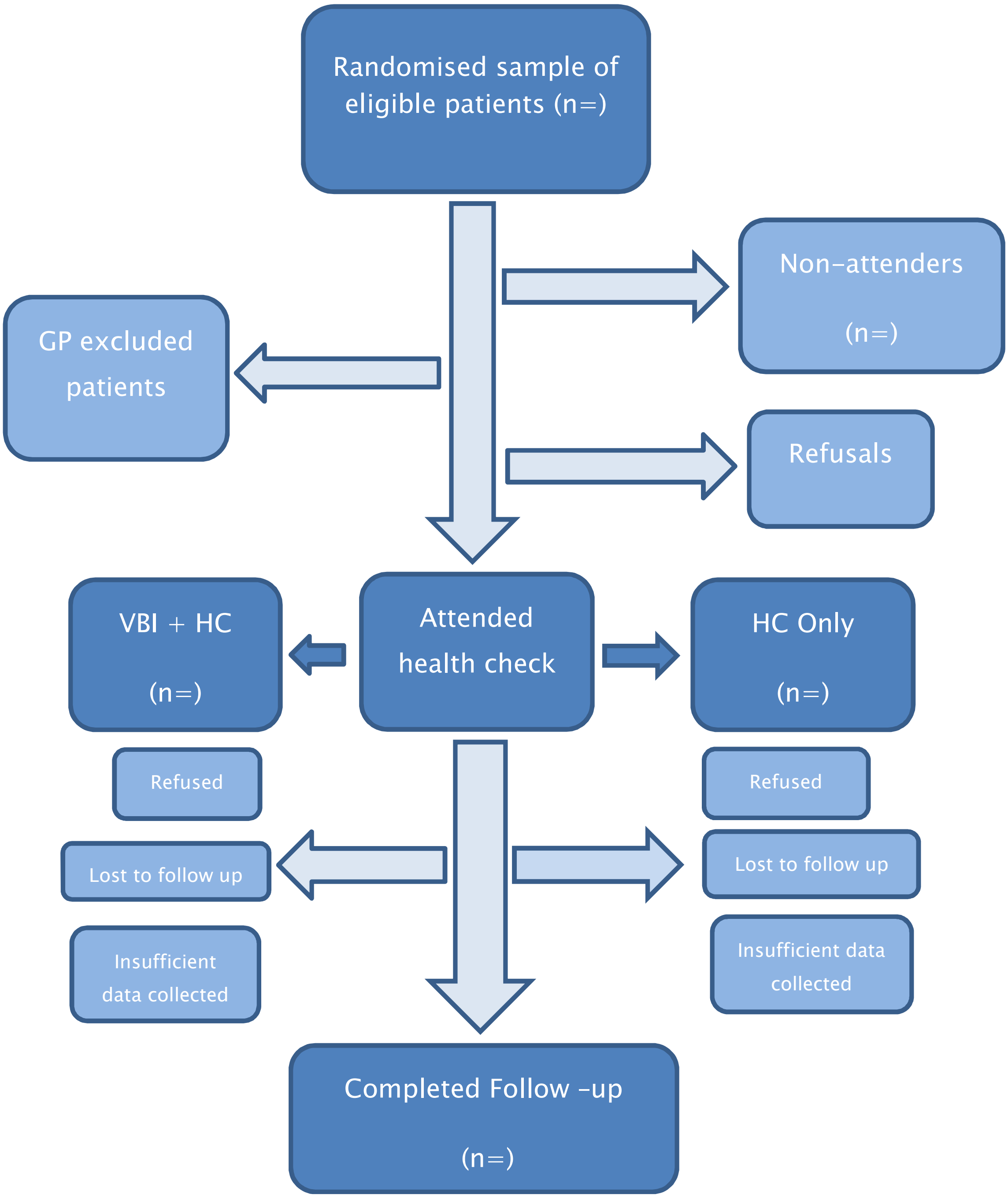

9.2 Baseline comparability of randomised groups

The initial tables will show the comparability of the groups at baseline. As the study is randomised, any observed differences are due to chance (assuming no failure of the randomisation). Therefore the null hypothesis of no population difference is known to be true, and p-values will not be used as these are not useful in this situation where the truth is known.

9.3 Comparison of rates of withdrawal and losses to follow up

Withdrawals and losses to follow-up will be summarised overall and within each arm. Comparisons between arms? will be made using the chi-squared test without continuity correction. Where indicated, Fisher's exact test will be used instead. Corresponding methods will be used to estimate 95% confidence intervals for overall and within-arm population rates.

9.4 Comparison of those followed-up with those lost to follow up

Those lost to follow-up (notably on the primary outcome) will be compared to those followed up in terms of baseline characteristics including gender, age, and employment status in order to characterise them. Univariate methods will be used such as the chi-squared test or the unpaired t-test or a non-parametric equivalent. Predictors of dropout will also be explored using logistic regression, extended to interact covariates with arm to investigate whether factors affecting dropout have a differential emphasis by arm. Any unexpected effects will be incorporated into the sensitivity analysis but not to alter the main pre-specified analysis.

10 Statistical Analyses

Linear and logistic regression, adjusting for the general practice stratifier (for continuous outcomes) and the pre-specified covariates (gender, five-year age-group), will be used to provide adjusted estimates of intervention effect. The statistics reported will be adjusted odds ratios, difference in arithmetic means, and ratio of geometric means. 95% confidence intervals and p-values will be reported for the intervention effect on each outcome.

10.1 Primary Efficacy Analysis

The primary efficacy analysis is a linear regression of accelerometer-derived step counts per minute, with adjustment for general practice, gender and five-year age-group.

10.2 Secondary Efficacy Analyses

The main secondary efficacy analysis is a sensitivity analysis to examine the robustness of the main analysis result to the 'missing at random' (MAR) assumption. The aim is to adequately explore the impact of departures from the MAR assumption on the primary outcome results [13].

For the sensitivity analysis, we pre-specify a range for counts per minute from -50cpm to +50cpm over which the mean of the "unobserved outcome data" in an arm might *depart* (or be different) from the mean of the "observed outcome data" in that arm. In other words, this range can be thought of as representing how much a typical subject with missing data may on average have had a different estimated treatment effect compared to having that of a corresponding subject with the outcome data observed (given the same baseline covariates and follow-up data in the linear model).

The range (-50cpm to +50cpm) is chosen to represent both negative and positive *departures* that could potentially arise as the "net effect" of alternative reasons which may be unknown. This range of 100 cpm (from -50cpm to +50cpm) is set this wide in order to explore sufficiently the sensitivity of the main results to departures from the MAR assumption. As a context, the approximate mean (and SD) in the pilot trial participants was 650cpm (SD 200cpm) and so the range of departures to be examined in these means spans a quarter of a SD in each direction amongst values of individuals.

At the end of the trial, the fractions of individuals with missing data for the primary outcome will be available in each arm as f_i (for intervention) and f_c (for control). The parameter which represents excess activity counts per minute in those with this missing compared to those with this observed, δ, will take values by passing across the range -50cpm to +50cpm. Three scenarios will be undertaken within the sensitivity analysis. These reflect whether departures from the MAR assumption apply

within the intervention arm only (pedometer intervention), within the control arm only (no intervention), or within both arms equally and in the same direction (thereby potentially cancelling out across the sensitivity range, if the dropout rate were to be the same in both arms).

Scenario 1: the treatment effect from the LME model will be increased by $f_i\delta$
Scenario 2: the treatment effect from the LME model will be increased by $-f_c\delta$
Scenario 3: the treatment effect from the LME model will be increased by $(f_i-f_c)\delta$

An additional secondary efficacy analysis will be the per protocol (PP) analysis. This will involve repeating the primary outcome analysis within a restricted (per population) population. This will comprise all participants except those who are later found to have been ineligible or who have not received their randomised intervention. The per protocol analysis may not be reported, or only as a single confirmatory sentence, if there it shows low impact due to a small number of protocol deviations or little effect on intervention effectiveness and its significance. If there are sizeable or impactful protocol deviations then the PP analysis may be extended to explore secondary outcomes.

10.3 Economic Analyses

The analysis will report the cost per patient to the NHS and to society in each arm, and the metabolic equivalent hours of physical activity in each arm. The increment between each group will also be calculated and the incremental cost per incremental met-hour of physical activity gain calculated. Analysis of uncertainty will follow good practice recommendations (Drummond et al. 2005), reporting standard errors around incremental costs and outcomes, and uncertainty in cost-effectiveness represented with the cost-effectiveness acceptability curve (showing the probability of cost-effectiveness as a function of willingness to pay for a met-hour improvement in physical activity).

The short-term within-trial effectiveness measures (met-hours) and costs will be combined with prior information and input into a previously developed decision model (GC V et al. manuscript in preparation) to estimate longer term costs and outcomes (measured in Quality Adjusted Life Years gained, QALYs), and cost-effectiveness (incremental cost per QALY gained). Analysis of uncertainty will be

reported as per the within-trial analysis (standard errors around increments and cost-effectiveness acceptability curves).

Reporting of the economic analyses will be consistent with current good practice recommendations (Husereau, Drummond et al. BMJ 2013).

11 Figures

Graphs will be used a part of the data checking process. This will include histograms, boxplots and scatterplots.

12 Reporting Conventions

P-values will be reported to two significant figures, but not more than 3 decimal places. P-values lower than 0.001 will be reported $p<0.001$. Outcomes will generally be reported to 3 significant figures, but outcomes which are related will be reported to the same number of decimal places (e.g. Physical activity energy expenditure domain outcomes).

13 Technical Details

SPSS version 22.0 (or a later version if available) will be used for the data checking and analyses.

14 References

[1] Department of Health. Putting Prevention First – NHS Health Check: Vascular Risk Assessment and Management Best Practice Guidance, 2009. http://webarchive.nationalarchives.gov.uk/20130107105354/http://www.dh.gov.uk/en/Publicationsandstatistics/Publications/PublicationsPolicyAndGuidance/DH_097489

[2] Health Survey for England 2012: Is the adult population in England active enough? Initial results. www.hscic.gov.uk/pubs/hse12early

[3] Craig R, Mindell J, Hirani V. (Eds). Health Survey for England 2008: Physical activity and fitness, Leeds: The Information Centre for Health and Social Care; 2009.

[4] National Institute for Health and Clinical Excellence. Four commonly used methods to increase physical activity: brief interventions in primary care, exercise referral schemes, pedometers and community-based exercise programmes for walking and cycling. Public Health Intervention Guidance no. 2. London: National Institute for Health and Clinical Excellence; 2006.

[5] Eden K, Orleans T, Mulrow C, Pender N, Teutsch S. Does counselling by physicians improve physical activity? A summary of the evidence for the US Preventative Services Task Force. Ann Intern Med 2002; 137:208.

[6] Greaves CJ, Sheppard KE, Abraham C, Hardeman W, Roden M, Evans PH, Schwarz P on behalf of the IMAGE Study Group. Systematic review of reviews of intervention components associated with increased effectiveness in dietary and physical activity interventions. BMC Public Health 2011; 11:119.

[7] Michie S, Richardson M, Johnston M et al. The Behaviour Change Technique Taxonomy (v1) of 93 hierarchically clustered techniques: Building an international consensus for the reporting of behaviour change interventions. Annals of Behavioral Medicine 2013; 1-15.

[8] National Institute for Health and Clinical Excellence. Physical Activity: brief advice for adults in primary care. Public Health Guidance 44. May 2013.

[9] McPhail S, Schippers M. An evolving perspective on physical activity counselling by medical professionals. BMC Family Practice 2012; 13-31.

[10] Moyer VA on behalf of the US Preventive Services Task Force. Behavioural Counselling Interventions to promote a Healthful Diet and Physical Activity for Cardiovascular Disease Prevention in Adults: U.S. Preventive Services Task Force Recommendation Statement. Ann Intern Med 2012; 157:367-372.

[11] Public Health England: NHS Health Check implementation review and action plan. July 2013

[12] White IR, Horton NJ, Carpenter J, Pocock SJ. Strategy for intention to treat analysis in randomised trials with missing outcome data. BMJ 2011; 342:d40.

[13] White IR, Carpenter J, Horton NJ. Including all individuals is not enough: lessons for intention-to-treat analysis. *Clin Trials* 2012; 9:396-407.
