# Supplementary material for: Evaluation of a very brief pedometer-based physical activity intervention delivered in NHS Health Checks in England: The VBI randomised controlled trial
Source: PLoS Med. 2020 Mar 6;17(3):e1003046. doi: 10.1371/journal.pmed.1003046 (PMC7059905; doi:10.1371/journal.pmed.1003046)
Supplement: S2 Text — (DOCX) [file pmed.1003046.s002.docx]

**S2 Text Supporting Information**

**Evaluation of a very brief pedometer-based physical activity intervention delivered in NHS Health Checks in England: The VBI randomised controlled trial**

# Deviation from protocol

We originally intended to report incremental cost per incremental MET (Metabolic Equivalent of Task)-hour of activity gained.[1] However, due to uncertainties over the algorithm to convert accelerometer counts to MET-hours,[2] we opted to present incremental cost per incremental 1000 steps increase per day. The main paper presents data on accelerometer counts as well as all other outcomes, allowing calculation of other incremental cost-effectiveness ratios (ICERs) as desired.

# Within-trial cost-effectiveness analysis

## Economic analysis

We performed within-trial cost-effectiveness analysis of the ‘Step It Up’ intervention compared with the Health Check alone from the perspectives of the NHS and society, examining the incremental cost per additional 1000 step counts. The price year of the study was 2014/15. We did not apply discounting because the time horizon of the analysis was less than one year.

Results are reported as point estimate cost and outcome (step counts) per patient in each group, increments and ICERs from the perspectives of the NHS and society. We performed bivariate regression analyses of costs and step counts, adjusting for five-year age group, sex and general practice. The regression coefficients were used to estimate the ICER, that is the mean difference in costs divided by mean difference in step counts between the groups multiplied by 1,000. To capture the uncertainty around the mean point estimates, we used non-parametric bootstrap (10,000 iterations). These 10,000 cost and effect pairs were plotted on a cost-effectiveness plane.

## Intervention costs

The cost of the ‘Step It Up’ intervention was calculated using data on time spent by the practice nurse or healthcare assistant in delivering the intervention. Cost per minute of face-to-face contact for practitioner was based on figures from the PSSRU unit costs [3], taking the midpoint of the relevant scales and including employer costs and appropriate overheads. We added costs for the pedometer, pedometer booklet and step chart (Table 1).

## Resource use and cost

We prospectively collected data on the use of NHS facilities, any out of pocket expenditures and work productivity alongside the ‘Step It Up’ intervention at 3 months follow-up. This included all face-to-face, home visits and telephone consultations with general practitioners (GPs), practice nurse and other health care staff, use of hospital outpatient facilities, attendance at hospital accident and emergency (A&E) facilities, number of hospital day cases and number and duration of hospital stay. We also collected data on participant expenditure on health, sports clubs or other physical activities. We used the work productivity and physical activity impairment (WPAI) questionnaire [4] to capture work productivity and time off work due to illness. All the resource uses were divided into four categories: NHS, social services, out-of-pocket expenditure and lost productivity.

**Table 1: Unit costs and source of unit costs applied to economic data**

| Resource use | Unit cost | Source |
| --- | --- | --- |
| Intervention cost |  |  |
| Face-to-face consultation (5 min nurse time) | £4.67 | PSSRU 2014-15 [3] p. 174 |
| Pedometer | £11.25 | VBI Study |
| Pedometer booklet | £1.52 | VBI Study |
| Step chart | £0.60 | VBI Study |
| Total intervention cost | £18.04 |  |
| NHS costs |  |  |
| GP surgery consultation | £44.00 | PSSRU 2014-15 [3] p. 177, per surgery consultation |
| GP home visit | £116.30 | PSSRU 2012-13 [5] p. 191 per out of surgery visit inflated to 2015 prices using HCHS index (PSSRU 2014-2015 [3] p. 242) |
| GP telephone consultation | £27.00 | PSSRU 2014-15 [3] p. 177, per surgery consultation lasting 7.1 min incl. direct care staff costs and qualifications |
| Nurse surgery consultation | £14.47 | PSSRU 2014-15 [3] p. 174, per hour of face-to-face contact including qualification x 15.5 min per surgery consultation |
| Nurse home visit | £56.00 | PSSRU 2014-15 [3] p. 174, per hour of face-to-face contact including qualification (assumes home visit takes 1 hr) |
| Nurse telephone consultation | £14.47 | Assumed same as surgery consultation |
| Specialist nurse surgery consultation | £25.00 | PSSRU 2014-15 [3] p. 175, per surgery consultation (15 mins) including qualifications |
| Physiotherapist surgery consultation | £47.95 | PSSRU 2012-13 [5] p. 175, mean cost for one-to-one contact inflated to 2015 prices using HCHS index (PSSRU 2014-2015 [3] p. 242) |
| Physiotherapist home visit | £47.95 | Assumed same as surgery consultation |
| Other AHP surgery consultation | £47.95 | Assumed same as surgery consultation |
| Other AHP home visit | £47.95 | Assumed same as surgery consultation |
| Other AHP telephone consultation | £23.97 | Assumed half of surgery consultation |
| Chiropractor surgery consultation | £55.00 | Mean cost of chiropractor consultation, http://chirocentre.co.uk/chiropractic-chiropractor-nhs/ |
| Chiropodist surgery consultation | £41.83 | PSSRU 2012-13 [5] p. 178, mean cost for a contact in chiropody/podiatry services inflated to 2015 prices using HCHS index (PSSRU 2014-2015 [3] p. 242) |
| Osteopathic consultation | £41.83 | Assumed same as chiropodist |
| Community nurse/midwife consultation | £27.92 | PSSRU 2014-15 [3] p. 169, assumed same as community nurse, 25 min consultation including qualification |
| Health visitor surgery consultation | £25.33 | PSSRU 2014-15 [3] p. 171, per hour of patient related work including qualification- assumed patient contact time of 20 min |
| Social worker office visit | £79.00 | PSSRU 2014-15 [3] p. 188, per hour client related work including qualification |
| Cognitive behaviour therapy | £98 | PSSRU 2014-15 [3] p. 90, per session |
| Outpatient appointment | £114.50 | Reference costs 2014-2015, weighted average of all outpatient attendance |
| Hospitalisation | £3,573.02 | Reference costs 2014-2015, weighted average of all elective inpatient stays |
| Day case procedure | £720.78 | Reference costs 2014-2015, day cases HRG data, worksheet DC, weighted average |
| A&E attendance | £131.92 | Reference costs 2014-2015, worksheet EM, weighted average of all A&E attendance |
| Travelling costs | £0.45 | HMRC [6], cost of car transport per mile |
| Cost of mammogram | £48.23 | Robertson et al [7], inflated to 2015 prices using HCHS index (PSSRU 2014-2015 [3] p. 242) |
| Time off work (hourly wage) | £14.08 | ONS Annual Survey of Hours and Earnings, 2015 [8] |

*A&E* accident and emergency, *AHP* allied health professionals, *GP* general practitioner, *HCHS* Hospital and Community Health Service, *HMRC* HM Revenue and Customs, *ONS* Office for National Statistics, *PSSRU* Personal Social Services Research Unit

Quantities of resources (NHS and social care services resources) used were multiplied by unit costs extracted from the standard UK sources (Table 1) and summed to generate total cost per participant. Lost productivity was measured in terms of wages forgone by multiplying the UK national median hourly wage rate [8] and the number of hours reported as taken off work by an individual.

# Results

## Data availability

Of the 1,007 participants randomised, 864 participants returned the completed resource use questionnaire at 3 months follow-up. In order to estimate the cost-effectiveness, we required complete cost and outcome data at 3 months follow-up. Of the 859 participants who provided valid data on the primary outcome at 3 months follow-up, resource use data was available for 856 (99.6%) participants. We used the complete case dataset to estimate the incremental cost per 1,000 additional step counts.

## Cost analysis

The ‘Step It Up’ intervention group participants received very brief advice on physical activity including pedometer, pedometer booklet and step chart. This costs £18.04 per person and includes a face-to-face nurse consultation. Healthcare resource use per participant was broadly similar between intervention and usual care groups (Table 2).

The primary care costs included GP surgery/home/phone and practice nurse surgery/home/phone consultations. Hospital costs included inpatient stays, outpatient visits and A&E attendance. Inpatient stays were costs as per episode.

**Table 2: Summary costs**

|  | **N (Intervention, usual care)** | **Intervention mean (SD)** | **Usual care mean (SD)** | **Unadjusted increment mean (SE)** | **Adjusted increment mean (SE)** |
| --- | --- | --- | --- | --- | --- |
| Cost of intervention | (416, 440) | 18.04 (0.00) | 0.00  (0.00) | 18.04  (0.00) |  |
| Primary care | (403, 430) | 32.05  (52.46) | 35.20 (60.82) | -3.14  (3.95) |  |
| Hospital costs | (402, 427) | 71.32  (370.68) | 68.93  (307.71) | 2.39  (23.61) |  |
| Total NHS costs | (416, 440) | 118.01  (379.22) | 101.29  (326.96) | 16.72  (24.16) | 21.55  (24.21) |
| Patient out-of-pocket costs | (416, 440) | 110.54  (194.80) | 101.12  (215.20) | 9.42  (14.06) |  |
| Lost productivity costs | (416, 440) | 386.03  (1130.44) | 358.10  (938.17) | 27.93  (70.85) |  |
| Total societal costs | (416, 440) | 614.58  (1212.01) | 560.51  (1042.17) | 54.07  (77.13) | 53.46  (76.97) |

Data reported are complete case analysis (means and unadjusted increment), and adjusted for sex, five-year age group and general practice.

## Incremental cost per additional step counts

When the cost of the ‘Step It Up’ intervention was added to NHS healthcare costs, total NHS costs were higher in the intervention group. When compared with usual care group, the incremental cost of the ‘Step It Up’ intervention was £16.72 (95% CI: -31 to 64, p=0.49). Likewise, total societal costs which included NHS healthcare, out-of-pocket and lost productivity costs were higher in the intervention group with an incremental cost of £54.07 (95% CI: -97 to 205, p=0.48) when compared to usual care group.

This produced an ICER of £96.32 per 1,000 steps/day using the NHS costs and £238.89 per 1,000 steps/day using the societal perspective, respectively, both compared to usual care (Table 3).

**Table 3: Cost-effectiveness analysis from the NHS and societal perspectives**

| **Analysis** | **Incremental cost** | **Incremental step counts** | **ICER** |
| --- | --- | --- | --- |
| NHS perspective | £21.55  (-26 to 69) | 224  (-193 to 640) | £96.32  (-996 to 1168) |
| Societal perspective | £53.46  (-98 to 205) | 224  (-193 to 640) | £238.89  (-2784 to 3102) |
| All values are bootstrapped (n=10,000 replications) mean and 95% Credible Interval (CrI). Incremental step counts differ from those reported in main manuscript Table 2 due to inclusion here of observations with complete cost and outcomes data only (n= 440, 416), compared with (442, 417) for which outcomes data were available). | | | |

## Analysis of uncertainty

Fig 1 shows the spread of bootstrapped 10,000 paired estimates of incremental costs and step counts on the cost-effectiveness plane. The majority of cost-effect pairs are located in the North East quadrant: 69% while using NHS perspective and 65% while using societal perspective.

| (a)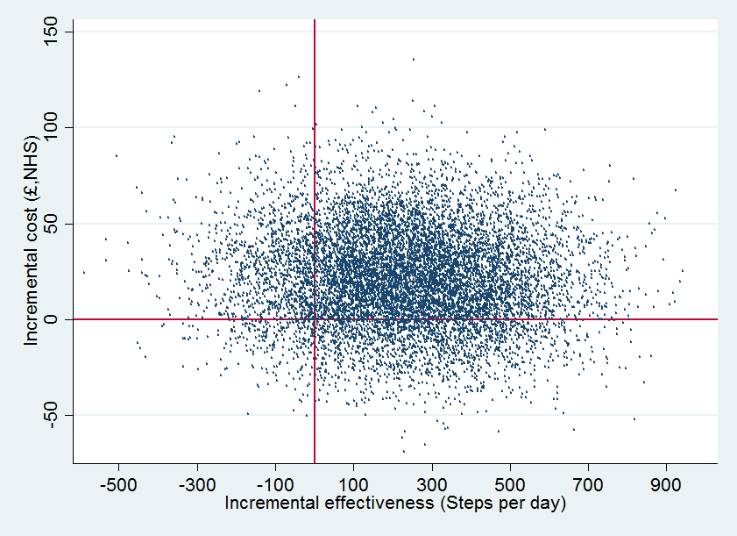 | (b)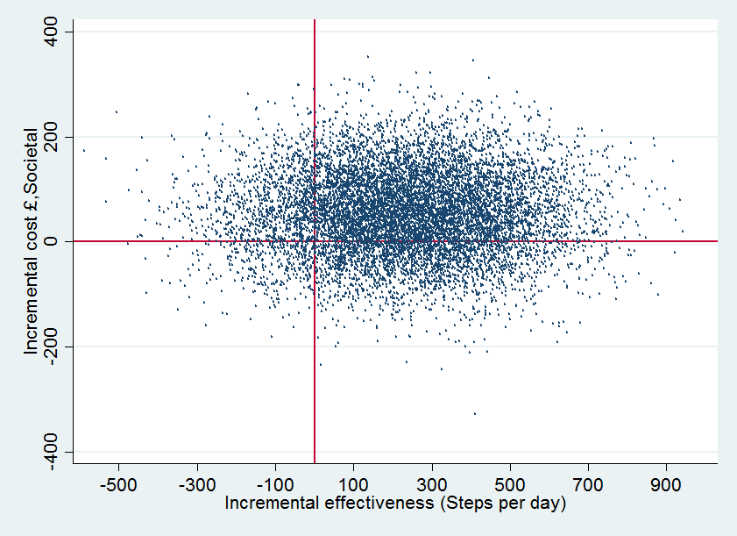 |
| --- | --- |

**Fig 1: Bootstrap results on the cost-effectiveness plane (based on 10,000 replications) using (a) NHS perspective, (b) Societal perspective**

The cost-effectiveness acceptability curve (CEAC) shows the probability that ‘Step It Up’ intervention is cost-effective for a range of values for the cost-effectiveness threshold from the perspectives of the NHS and society (Figure 2). The likelihood that the ‘Step It Up’ intervention is cost-effective rises to 80% when society is willing to pay £500 for 1000 additional steps. When considering the NHS perspective, the probability that the intervention would be cost-effective is 84% at a willingness to pay threshold of £1150.


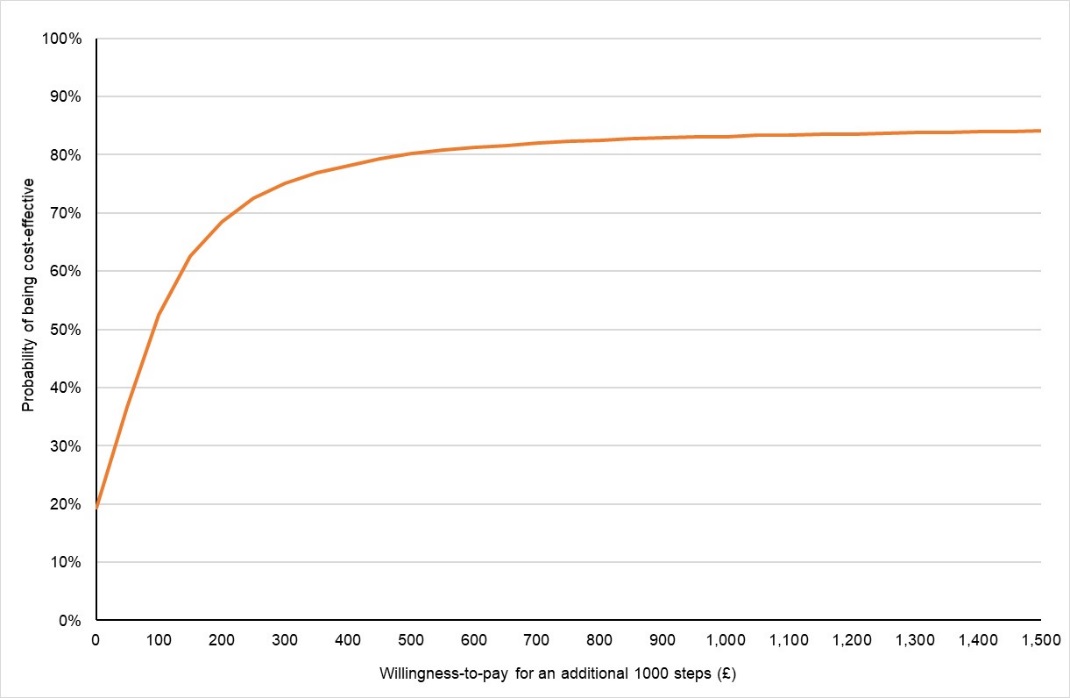


**Figure 2: Cost Effectiveness Acceptability Curve (using the NHS perspective)**

## Work productivity

On average, 59% participants in the intervention group and 62% in the usual care group were in work. Among those employed, intervention group participants reported slightly higher impairment while working (presenteeism) and overall work impairment scores (Table 4). However, between- group differences in work productivity and impairment scores were not statistically significant.

**Table 4: Mean work productivity and activity impairment scores by study groups**

|  | **N (intervention, usual care)** | **Intervention mean (SD)** | **Usual care mean (SD)** | **Difference (95% CI)** |
| --- | --- | --- | --- | --- |
| % work time missed due to health (Absenteeism) | (214, 233) | 0.9% (6.7) | 1.14% (9.3) | -0.22%  (-1.75 to 1.31) |
| % impairment while working due to health (Presenteeism) | (221, 249) | 5.9% (13.9) | 4.9% (11.3) | 1.07%  (-1.2 to 3.4) |
| % overall work impairment due to health (Productivity loss) | (210, 229) | 6.7% (15.9) | 5.0% (11.5) | 1.69%  (-0.9 to 4.28) |
| % activity impairment due to health (Activity impairment) | (390, 421) | 11.8% (21.7) | 12.7% (23.2) | -0.9%  (-4.0 to 2.22) |

# References

[1] Mitchell J, Hardeman W, Pears S, et al. Effectiveness and cost-effectiveness of a very brief physical activity intervention delivered in NHS Health Checks (VBI Trial): study protocol for a randomised controlled trial. Trials 2016;17:303.

[2] Harrington DM, Welk GJ, Donnelly AE. Validation of MET estimates and step measurement using the ActivPAL physical activity logger. J Sports Sci 2011;29:627-33.

[3] Curtis L, Burns A. Unit Costs of Health and Social Care 2015. Canterbury: Personal Social Services Research Unit, University of Kent, 2015.

[4] Reilly MC, Zbrozek AS, Dukes EM. The validity and reproducibility of a work productivity and activity impairment instrument. Pharmacoeconomics 1993;4:353-65.

[5] Curtis L. Unit Costs of Health and Social Care 2013. Canterbury: Personal Social Services Research Unit, University of Kent, 2013.

[6] HMRC. Rates and allowances: travel - mileage and fuel allowances. Available from: https://www.gov.uk/government/publications/rates-and-allowances-travel-mileage-and-fuel-allowances [Accessed 22 April 2016].

[7] Robertson C, Arcot Ragupathy SK, Boachie C, et al. The clinical effectiveness and cost-effectiveness of different surveillance mammography regimens after the treatment for primary breast cancer: systematic reviews registry database analyses and economic evaluation. Health Technol Assess 2011;15:v-vi, 1-322.

[8] Office for National Statistics. Annual Survey of Hours and Earnings: 2015 Provisional Results. Available from: http://www.ons.gov.uk/employmentandlabourmarket/peopleinwork/earningsandworkinghours/bulletins/annualsurveyofhoursandearnings/2015provisionalresults [Accessed 25 April 2016].
