# Supplementary material for: Evaluation of a very brief pedometer-based physical activity intervention delivered in NHS Health Checks in England: The VBI randomised controlled trial
Source: PLoS Med. 2020 Mar 6;17(3):e1003046. doi: 10.1371/journal.pmed.1003046 (PMC7059905; doi:10.1371/journal.pmed.1003046)
Supplement: S2 Table — (DOCX) [file pmed.1003046.s004.docx]

**S2 Table**: Sensitivity analysis: Baseline characteristics in completers (n=859) by arm, their contribution to further explaining the primary outcome correlation, and their impact on the intervention effect

| Characteristic | Control  (*n*=442) | Intervention (*n*=417) | Partial  correlation† | Intervention effect  after adjustment†† |
| --- | --- | --- | --- | --- |
| Mean (SD) age | 56.7 (9.3) | 56.6 (9.5) | Already included | Unchanged |
| Female | 61% | 62% | Already included | Unchanged |
| White ethnicity | 96% (420/440) | 96% (401/416) | 0.014 | 8.6 (-18.9, 36.2) |
| Married or cohabiting | 80% (330/411) | 80% (320/398) | 0.012 | 9.1 (-18.5, 36.6) |
| Have dependants | 33% (137/411) | 38% (153/399) | 0.050 | 8.7 (-18.9, 36.3) |
| Work status: paid work | 59% (246/415) | 59% (236/399) | 0.113 | 8.1 (-19.3, 35.5) |
| Income ≤£30,999 | 49% (174/358) | 42% (148/351) | 0.100 | 11.5 (-16.1, 39.0) |
| Non-manual occupation | 70% (179/255) | 67% (169/250) | 0.234 | 6.4 (-20.4, 33.1) |
| Qualification: none or GCSE | 70% (298/425) | 76% (312/409) | 0.052 | 7.6 (-20.0, 35.3) |
| Accommodation: ownership | 86% (350/408) | 89% (356/400) | 0.054 | 8.9 (-18.7, 36.5) |
| Car ownership | 94% (386/412) | 95% (382/401) | 0.040 | 10.0 (-17.6, 37.5) |
| Median IMD deprivation | 10.9 | 10.8 | 0.013 | 9.1 (-18.5, 36.6) |
| Median CVD risk score | 6.6% | 6.7% | 0.021 | 8.8 (-18.7, 36.4) |
| GPPAQ: Moderately active /Active | 68% | 72% | 0.199 | 5.2 (-21.7, 32.2) |

† The partial correlation represents the correlation of the characteristic with the primary outcome over and above having adjusted for sex, age, and practice. It was obtained as the multiple correlation between the residuals from the primary outcome model (adjusting for sex, age and practice) and each baseline characteristic in its categorical form including a category for missing data.

†† The intervention effect was estimated to be 8.8 (95%CI: -18.7 to 36.3) from the primary outcome model which a priori adjusted for sex, age and practice. As age and sex were already included, the intervention effect was unchanged. The intervention effect was hardly changed from adjustment for the other factors (being estimated between 5.2 and 11.5) and so we did not further adjust for multiple characteristics together.
