# Supplementary material for: Evaluation of a very brief pedometer-based physical activity intervention delivered in NHS Health Checks in England: The VBI randomised controlled trial
Source: PLoS Med. 2020 Mar 6;17(3):e1003046. doi: 10.1371/journal.pmed.1003046 (PMC7059905; doi:10.1371/journal.pmed.1003046)
Supplement: Attachment — Submitted filename: Hardeman et al Response to reviewers.docx [file pmed.1003046.s005.docx]

**Response to Reviewers**

**Requests from the editors**

1. Our data policy calls for a primary non-author contact for inquiries about access to data, and so we ask you to remove the author contact details. Please adapt the data statement wording to "... will be made available upon reasonable request". It may be helpful to add additional institutional contact details at a future stage.

We have adapted the data statement, which now reads: “Anonymised patient level data will be made available on reasonable request to the University of Cambridge Primary Care Unit Secretariat ([PCUPA@medschl.cam.ac.uk](mailto:PCUPA@medschl.cam.ac.uk)) after submitting a proposal and signing a data access agreement. Consent for data sharing was not explicitly obtained from participants, but the data are anonymised and the risk of identification is low”.

2. It may be that the competing interest with AbbVie can be removed in view of the date: please consult our conflict of interest policy (<https://eur01.safelinks.protection.outlook.com/?url=https%3A%2F%2Fjournals.plos.org%2Fplosmedicine%2Fs%2Fcompeting-interests&amp;data=02%7C01%7Cw.hardeman%40uea.ac.uk%7Cf9c861e1c2854645cc0b08d74d67b1ce%7Cc65f8795ba3d43518a070865e5d8f090%7C0%7C0%7C637062980074474198&amp;sdata=OYMgI%2BU%2Fs9kiOdf9LPnvbQthpggEZJy7pqBo%2FANG4ss%3D&amp;reserved=0>).

Thank you. We have checked the conflict of interest policy and removed the competing interest with AbbVie. The section now reads: “All authors have read the journal’s policy and have declared no competing interests exist”.

3. Please adapt the title to "Evaluation of a very brief ...".

We have changed the title in the manuscript and supplemental information S1 to: “Evaluation of a very brief pedometer-based physical activity intervention delivered in NHS Health Checks: The VBI randomised controlled trial”.

4. Please add an additional sentence or two to your abstract to summarize the secondary outcome findings.

We have added the following sentence in the abstract: “We found no significant between-group differences in the secondary outcomes of accelerometer-derived step counts per day and time spent in moderate to vigorous, vigorous and moderate intensity activity; and self-reported total physical activity, home-based, work-based, leisure based and commuting physical activity, and screen or TV time.”

5. We ask you to add a new final sentence of the "methods and findings" subsection of your abstract to summarize the study's main limitations.

We have added the following sentence at the end of the “methods and findings” subsection: “The study’s limitations include a participation rate of 16% and low return of audiotapes by practices for fidelity assessment.”

6. Please refer to the attached CONSORT document at a suitable point in your methods section. We ask you to include the study analysis plan as a supplementary document if possible, and again refer to this in the main text. Please highlight any analyses that were not prespecified in the main text (including that noted in the supplementary file).

We assume that you refer to the CONSORT checklist which is supplemental information S2. We have added the following reference to S2 in the statistical analysis section: “We conducted our analyses according to CONSORT guidelines (see S2 – supporting information), and agreed the analysis plan a priori with the independent Trial Steering Committee”. We have enclosed the statistical analysis plan as S1 and referred to it in section ‘’Randomisation and masking’ in the methods. We have highlighted two non pre-specified subgroup variables in Table 3 and added a supplementary table S4.

7. At the start of the discussion section of your main text, "rigorously conducted" seems a little strident, and could be de-emphasized by being moved to the "strengths and limitations" section.

We have changed the first sentence in the discussion to: “Our trial showed no evidence for a positive effect of a five-minute pedometer-based intervention delivered as part of NHS Health Checks in primary care on objectively measured and self-reported physical activity after three months, compared to the Health Check alone.”

8. There may be an indication in figure 1 and elsewhere of greater attrition in the intervention arm, and we suggest addressing this in the "strengths and limitations" section.

Thank you for this observation. Attrition was higher in the intervention arm, and we do not know the reason as the main difference between the two arms is in ‘no reason given’. Overall, retention rates were high at 85% and any differences between those lost to follow-up and those who completed follow-up did not influence the main result as shown in new supplementary table S4. We have added the following sentence in the ‘strength and limitations’ section: “Attrition was low overall, slightly greater in the intervention arm but was not seen to influence the main finding.”

9. Please substitute "sex" for "gender" as appropriate throughout the text.

Thank you, we have done this in the manuscript and S3 supporting information.

10. Throughout the text, please adapt reference call-outs to the following style: "... per day [11,12].".

We have done this.

11. Please review the reference list to ensure that journal names are abbreviated consistently (e.g., "PLoS Med." for reference 27).

We have reviewed the reference list and edited references accordingly.

12. Please adapt your attached CONSORT checklist so that individual items are referred to by section (e.g., "Methods") and paragraph number rather than by line or page numbers, as the latter generally change upon publication.

We have adapted the CONSORT checklist as suggested and added headings of paragraphs as appropriate.

13. As the trial protocol is published, you may wish to remove the attached document.

We have removed the trial protocol as an attachment.

**Reviewer #1 (statistical reviewer)**

We were pleased to read that the reviewer believed that our trial was well-conducted and that that our reporting was mostly of a good standard.

1) On page 17, it would be good to provide a table on comparisons between patients with complete data and those with missing data, maybe as supplementary tables.

Thank you. We have retained most of the information in the text: “Compared to those with missing primary outcome data, participants who completed follow-up with primary outcome had higher modelled 10-year cardiovascular disease risk (median 6.7% (IQR 2.7%, 11.5%) vs 4.9% (1.9%, 8.7%), *p*=0.005), were less likely to be in paid employment (59% vs 75%, *p*<0.001) and manual workers (25% vs 41%, *p*=0.002), and were older (56.6 (*SD*=9.4) vs 52.9 (*SD*=9.4).” Furthermore, we have referred to table S4 comparing the baseline characteristics between arms among those with complete data.

2) Table 1. Could authors please put a percentage sign (%) for all the percentages in the table?

Thank you, we have made the suggested change in Table 1. We have done this in Table 5 too.

3) The first paragraph on page 20 provides key results for the paper including ITT analysis, sensitivity analysis and PP analysis. Could authors please highlight these analyses (maybe use subtitles) and also make it very clear whether these results are significant or not, therefore deliver a clear message on whether these analyses changed results in any way. Also, for sensitivity and PP analyses could authors please provide complete results as supplementary tables?

Thank you. We have added subtitles under a new main heading “Sensitivity analyses”: Sensitivity of the result to missing data, Sensitivity of the results to missing at random assumption, and Per-protocol analysis. We have clarified the non-significant results from these analyses and provided complete results of the PP analysis in these sections.

4) In table 2, the primary outcome analysis was only adjusted for age, gender and practice however there are quite a few other variables in the baseline which could potentially be adjusted such as Physical activity status and multiple deprivation (IMD). Would adjusting for more baseline variables change the final results in any way?

Thank you. We adjusted for age, gender and practice because these covariates were pre-specified in the Statistical Analysis Plan. The primary outcome result was clearly non-significant (8.8 (95% CI: -18.7 to 36.3)) with p-value 0.53. However, in the new table S4 in the Appendix, we have further adjusted for the other baseline characteristics including physical activity status and IMD. These characteristics were quite well balanced between arms in the completers and had quite low correlation with the primary outcome after having adjusted for the pre-specified covariates. Consequently, the additional adjustment for each of these characteristics did not change the intervention effect or confidence interval very much at all, and the conclusions from this sensitivity analysis were the same as those for the main analysis.

**Reviewer #2**

We were pleased that the reviewer felt that our pragmatic trial addressed an important question and that it makes an important contribution to the evidence base.

My only concern is the statement "Fidelity of delivery was partial." in the abstract. I think this is potentially misleading given the information on page 26 that "491/505 participants received the 'Step It Up' intervention as planned." I understand that this statement is based on the assessment of program content but feel that this should be made clearer and both pieces of information should be presented in the abstract.

Thank you for this helpful comment. We agree that the abstract could be potentially misleading. Indeed, there are multiple indicators of intervention fidelity. We have reported two indicators: 1) the proportion of intervention participants receiving the planned intervention and 2) faithful delivery of intervention components including behaviour change techniques from an in-depth assessment of a subsample of audiotaped NHS health checks consultations. We have clarified this in the abstract as follows.

In the methods: “We conducted an in-depth fidelity assessment on a subsample of Health Check consultations.”

In the results: “97% (491/505) of intervention participants received the ‘Step it Up’ intervention. Analysis of 37 intervention consultations showed that 60% of ‘Step it Up’ components were delivered faithfully.”

**Reviewer #3**

We are pleased that the reviewer felt that our trial was novel, had potential for wide public health reach, and reported a good example of integrating behaviour change techniques in very brief interventions.

1. Most participants were already active at baseline (GPPAQ and report of control group's step count >8000 steps), and so it is unclear why they received an intervention designed for inactive people and were not screened out. A sub-group analysis on the inactive patients may be more appropriate to demonstrate the effectiveness of the intervention.

Thank you for this comment. Our participants were only slightly more active than those in the PACE trials. We considered physical activity measurement prior to the Health Check. Screening out potential participants who were too active would have required eligibility checking prior to randomisation at the start of the Health Check consultation. Randomisation had to happen at the start of the Health Check, as it was up to the health practitioner to deliver ‘Step it Up’ during or after the routine Health Check.

This was a pragmatic trial in which participants were invited to our trial at the same time as their invitation to the NHS Health Check, which is routine practice. In principle eligibility in terms of physical activity could have been assessed at three time points, which we considered during trial design, but none of them were feasible.

The first option could have been to use physical activity data from practice records. However, no consistent data on physical activity levels are available which could be used to screen out people at the point of invitation.

The second option would have been to screen people after they had expressed an interest in taking part in our trial. As part of pilot work for the trial we explored the feasibility of baseline physical activity measurement at this point. The findings showed that this measurement reduced the uptake of the NHS Health Check, which was not acceptable to the practices as the Health Check is routine practice.

A third option would have been to screen people at the start of the Health Check, prior to consenting and randomising. Physical activity assessment, albeit with an imprecise measure, is a component of the Health Check and changing its sequence in the consultation would have changed routine practice. An additional screening assessment would have added valuable time during a busy clinic. Finally, self-reported physical activity is prone to error and bias, so we might have excluded potential participants who could have benefited from our intervention.

Nevertheless, we have added a further post-hoc subgroup analysis by self-reported activity, which shows no significant effect. We have reported this in Table 3.

We mention in the discussion in the section ‘Study strengths and limitations’ that it was not feasible and acceptable to conduct baseline measurement prior to the Health Check consultation. We have clarified that this included objective measurement of physical activity: “Our pilot work showed that baseline measurement, which included objectively measured physical activity, reduced NHS Health Check uptake, which was not acceptable to participating practices as they constitute routine care”. We have added two sentences in the section about limitations: “Physical activity screening during the Health Check prior to consenting and randomisation to exclude very active adults was not feasible as it would have added time and changed routine practice. Our participants were more active than might have been expected, potentially reducing room for improvement, although self-reported physical activity at baseline did not moderate any intervention effects.”

2. Author Summary statement - last point "Our general population findings do not imply that primary care practitioner should refrain from providing (very) brief PA advice opportunistically" has too many double negatives. Simply - Primary Care professionals should continue to providing very brief PA advice, opportunistically.

Thank you for this excellent suggestion; we have changed the author summary statement.

3. Given the focus on a pragmatic intervention and approach, one of the key findings should be in relation to the poor fidelity of the intervention - it is important to note that the intervention was not delivered as intended on this occasion. This is especially pertinent given the aspects which were not delivered relate to aspects expected to support behaviour change. The results of the fidelity test should be in the results section of the abstract prior to the sentence "There were no significant between-group differences in activity volume (8.8 counts per minute; 95% CI -18.7, 36.3)" in order to alert readers that they should interpret the lack of difference between groups with care.

Thank you for this comment, we agree that intervention fidelity is important and may partly explain the findings of the trial. We are cautious however as only 37/505 (7.3%) of intervention consultations were audio-recorded and thus assessed. We do not know whether these findings generalise to all intervention consultations.

We appreciate that whilst we reported this small sample size in the methods, this was not reported in the results. We have clarified this by editing the following sentence in the section about process evaluation: “An estimate of fidelity of intervention delivery based on an in-depth assessment of 37/505 (7.3%) audio-recorded consultations was 60%, meaning that, on average, 9 out of 15 components were delivered”. In response to the first comments by reviewer #2 we have also reported this more clearly in the abstract. Furthermore, we have added the following sentence in the summary statement and conclusions: “Despite the intervention being apparently simple and very brief, fidelity of delivery was suboptimal”.

On balance, we feel that it is best to report the primary and secondary outcomes first, then the health economic evaluation and finally all the process evaluation findings in one section to illuminate the findings from the outcome and health-economic evaluation.

4. Re: NHS Health Checks - would be useful to know how many people attend these across England, to better understand reach.

Thank you. We reported the proportion of people in England taking up an invitation in the discussion. We have updated this sentence with the latest available data from Public Health England. The sentence now reads: “During 2015 and 2019 47.4% of eligible people in England took up an NHS Health Check invitation”.

5. Although there is a protocol paper available - Information is insufficient to understand intervention - An additional sentence here would be useful - What goals were suggested? Were they individualised? Over how many weeks? Etc

Thank you for this comment. The health practitioners mentioned the recommendation of being physically active for 30 minutes on most days of the week or trying to accumulate 10,000 steps per day. They presented the 10,000 steps as a good target to aim for but encouraged participants to gradually increase their step goals each week towards the target if they were able to. Participants were given a Step Chart and encouraged to use this to set weekly step goals and record daily steps walked to review progress. The health practitioners did not suggest any specific activities, but mentioned that any activity which raises heart rate, makes you breathe a little more heavily, and results in a light sweat counts as physical activity.

We have added the following sentences at the end of the ‘Procedures’ section in the methods: “Participants were told that 10,000 steps per day was a good target to aim for but were encouraged to gradually increase their step goals each week if they were able to. They were given a Step Chart and encouraged to use this to set weekly step goals and record daily steps. The health practitioners did not suggest specific activities, but mentioned that any activity which raises heart rate, makes you breathe a little more heavily, and causes a light sweat counts as physical activity.”
