# Supplementary material for: Evaluation of a very brief pedometer-based physical activity intervention delivered in NHS Health Checks in England: The VBI randomised controlled trial
Source: PLoS Med. 2020 Mar 6;17(3):e1003046. doi: 10.1371/journal.pmed.1003046 (PMC7059905; doi:10.1371/journal.pmed.1003046)
Supplement: Attachment — Submitted filename: Response to Editors and Production Team R3.docx [file pmed.1003046.s006.docx]

**Response to Comments by Editors and Production Team**

Please find our response to your queries below.

TABLES and BOXES:

1. Tables 3 uses returns to control text wrapping. This is indicated by a [¶ or ↵] symbol when "Show Paragraph Marks" is turned on in Word. Please either split those cells into separate cells to achieve alignment or remove the returns.

We have done this and created an additional row.

ARTICLE FILE:

1. Please indicate the corresponding author in the byline by placing an asterisk (*) after their affiliation number.

We have done this.

2. Please include the corresponding author's email address on the title page of your manuscript, indicated by an asterisk (*). Only an asterisk and the email address itself are required.

We have done this.

SUPPORTING INFORMATION:

1. Please upload your supporting information as individual files, and use the following naming format: S1 Text; S1 Fig; S1 Table, S1 Data, etc. Each file type should start as S1 and increase numerically: S1 Text, S2 Text, etc. Note that all relevant in-text citations and captions will need to be updated accordingly.

We have done this.
